# Understanding the functionality of the rumen microbiota: searching for better opportunities for rumen microbial manipulation

**DOI:** 10.5713/ab.23.0308

**Published:** 2023-12-29

**Authors:** Wenlingli Qi, Ming-Yuan Xue, Ming-Hui Jia, Shuxian Zhang, Qiongxian Yan, Hui-Zeng Sun

**Affiliations:** 1 Key Laboratory of Dairy Cow Genetic Improvement and Milk Quality Research of Zhejiang Province, College of Animal Sciences, Zhejiang University, Hangzhou 310058, China; 2 CAS Key Laboratory of Agro-Ecological Processes in Subtropical Region, Hunan Provincial Key Laboratory of Animal Nutritional Physiology and Metabolic Process, Institute of Subtropical Agriculture, Chinese Academy of Sciences, Changsha 410125, China

**Keywords:** Functionality, Omics Technologies, Rumen Microbiota

## Abstract

Rumen microbiota play a central role in the digestive process of ruminants. Their remarkable ability to break down complex plant fibers and proteins, converting them into essential organic compounds that provide animals with energy and nutrition. Research on rumen microbiota not only contributes to improving animal production performance and enhancing feed utilization efficiency but also holds the potential to reduce methane emissions and environmental impact. Nevertheless, studies on rumen microbiota face numerous challenges, including complexity, difficulties in cultivation, and obstacles in functional analysis. This review provides an overview of microbial species involved in the degradation of macromolecules, the fermentation processes, and methane production in the rumen, all based on cultivation methods. Additionally, the review introduces the applications, advantages, and limitations of emerging omics technologies such as metagenomics, metatranscriptomics, metaproteomics, and metabolomics, in investigating the functionality of rumen microbiota. Finally, the article offers a forward-looking perspective on the new horizons and technologies in the field of rumen microbiota functional research. These emerging technologies, with continuous refinement and mutual complementation, have deepened our understanding of rumen microbiota functionality, thereby enabling effective manipulation of the rumen microbial community.

## INTRODUCTION

The rumen microbiome is indispensable for the survival, productivity, and overall health of ruminant animals, as the microbiome plays a vital role in their early development, health, and physiological processes. The diversity of rumen microorganisms results from mutual selection and coevolution between the microorganisms and their hosts, resulting in a dynamic balance of interdependence and restriction [1]. On the one hand, the host provides a favorable living environment and fermented substrates for the growth of rumen microorganisms [2]. On the other hand, rumen microbes play a crucial role in the breakdown of plant cellulose, hemicellulose, starch, and other components, providing energy and essential nutrients to the host for survival and production. Therefore, investigating the types and functions of rumen microorganisms, as well as the interactions between these microorganisms and their host, holds great significance in enhancing the production performance of ruminants.

**Figure 1:**
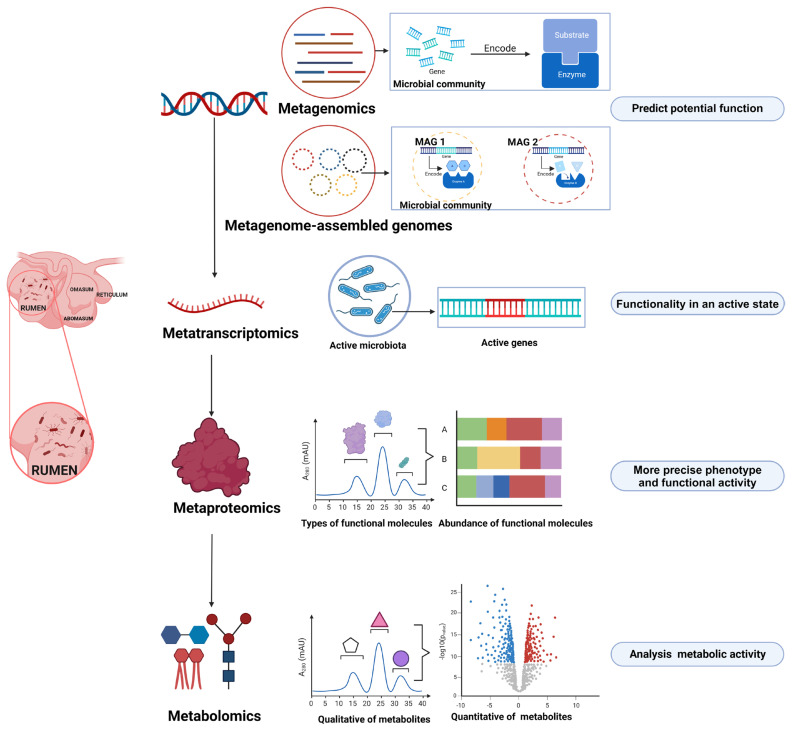
Omics technologies for investigating rumen microbial function (Created with BioRender.com).

For a long time, our knowledge on the rumen microbiome has relied primarily on iso lation and culture-based methods. Although this approach is limited when culturing the entire spectrum of rumen microbes, it can provide valuable insights into the accurate functional capacities of specific microbial members (Table 1). In recent years, however, researchers have focused more on omics-based approaches in rumen microbiology research (Figure 1) [3–5]. Through this method, microbial community phylogeny, diversity, composition, and functional capabilities can be explored in a culture-independent and high-throughput manner, which has significantly expanded our knowledge on the rumen microbiome and its ecological roles.

Studies have shown that while the composition of rumen microbial communities may vary across studies, the metabolic and functional aspects of these communities remain relatively stable [6]. This reveals a significant inconsistency between the current knowledge on rumen microbial taxonomy and functional annotations. There are two potential reasons for this discrepancy. First, there is a deviation between the principles of species taxonomy and functional classification. For example, two strains from the same species may perform distinct metabolic functions, while different functions may be shared by multiple microbial lineages [7]. Second, when translating species taxonomy annotations into metabolic functions, the redundancy and variability of these functions become evident [8]. Therefore, it is essential to recognize the limitations of focusing solely on species taxonomy [9]. Instead, attention should be directed toward the functional repertoire of the rumen microbiota to reveal the true events.

## RUMEN MICROBIAL SPECIES INVOLVED IN THE COMMON FUNCTIONS REVEALED BY CULTURE-BASED METHODS

### Hydrolysis of macromolecules

#### Microbial species related to polysaccharide degradation in the rumen

Among all livestock species, ruminant animals are the most efficient in utilizing fiber due to their unique digestive system, the rumen microbiota. The cellulolytic bacterium *Fibrobacter succinogenes* was initially identified in the rumen of cows in 1947 [10]. Moreover, by assessing the potential for cellulose degradation through enzyme catalysis in pure isolates and employing relative quantification real-time polymerase chain reaction (PCR), *F. succinogenes* was revealed as the predominant bacterium in terms of efficiency and prevalence in cellulose degradation within the rumen [11]. Another cellulolytic bacterium, *Ruminococcus flavefaciens*, exhibits comparable abundance to *F. succinogenes* in lactating cows under conventional feed conditions [12]. Under cellulose-restricted conditions, *R. flavefaciens* becomes dominant. The third most common group of cellulolytic bacteria is *R. albus*. Compared to *F. succinogenes* and *R. flavefaciens*, *R. albus* is less abundant, but it is particularly competitive in the initial adhesion to cellulose and growth [13]. Other cellulolytic bacteria have also been cultured and well studied. For example, *Clostridium lochheadii* [14] is highly active in digesting cellulose. *C. longisporum*, *Eubacterium cellulosolvens*, and *Butyrivibrio fibrisolvens* [15] are also cellulolytic bacteria isolated from the rumen.

In addition to bacteria, certain protozoa exhibit cellulolytic activity, which may arise from ingested fibrolytic microorganisms or their own fiber-degrading enzymes. Among the rumen protozoa studied, *Eudiplodinium maggii*, *Epidinium ecaudatum*, and *Ostracodinium dilobum* emerged as the most efficient cellulose degraders [11]. Nevertheless, exploring this capability is challenging due to the complexities associated with maintaining ruminal protozoa in culture [16]. Anaerobic fungi have also been successfully isolated from various ruminant species, showcasing their impressive capacity for cellulose hydrolysis [17]. Despite their relatively low numerical abundance, these anaerobic fungi play a significant role in cellulose degradation within the rumen [18].

Compared to cellulose, the structure of hemicellulose is more complex and easier to hydrolyze; thus, research on hemicellulose degradation is limited. All cellulolytic bacteria can degrade hemicellulose. In early experiments, in addition to cellulolytic bacteria, *Lachnospira multiparus*, and *Prevotella ruminicola* isolated and cultured from the rumen could degrade hemicellulose [19]. *Prevotella species*, although less efficient than *B. fibrisolvens* [20], play a crucial role in ruminal hemicellulose degradation due to their prevalence as very common microbes in the rumen. Notably, the degradation capabilities of different hemicellulolytic bacteria are specialized due to the different structures of hemicellulose. For example, *B. fibrisolvens* is more effective in degrading xylan than other hemicelluloses (xyloglucan, glucomannan, and β-glucan) [20]. The degradation of glucomannan is primarily carried out by *Streptococcus* species [20]. Three ruminal protozoa species (*Polyplastron multivesiculatum*, *Eudiplodinium maggii*, and *Entodinium* species) can also degrade hemicellulose due to their endo-xyloglucanase and endo-xylanase activities [21]. However, most of the discovery of fungi is based on metagenomics, which should be researched more through culture.

The rumen has a large population of amylolytic bacteria, such as *S. bovis*, *P. ruminicola*, *Ruminobacter amylophilus*, *Succinimonas amylolytica*, and *Selenomonas ruminantium* [22,23]. Notably, resistant starch is degraded by the specialized *R. bromii* [24]. Some cellulolytic bacteria can also utilize starch, including *B. fibrisolvens*, *F. succinogenes*, and *Clostridium* species [25,26]. Amylolytic bacteria breakdown starch into oligosaccharides, which are usually directly fermented into volatile fatty acids (VFAs). However, for some amylolytic bacteria, the fermentation end products are intermediate molecules. For example, *S. bovis* primarily produces lactate as the end product [23], while *R. amylophilus* produces succinate during amylolysis [22]. These lactate and succinate molecules subsequently undergo further fermentation by other bacteria into VFAs. Approximately 20% to 45% of starch degradation activity in the rumen is attributed to protozoa [27]. Protozoal species including *Eremoplastron bovis*, *Diploplastron affine*, *Ophryoscolex caudatus*, and *Polyplastron multivesiculatum* exhibit a remarkable capacity for starch degradation [28]. Additionally, rumen fungi like *Orpinomyces joyonii*, *Neocallimastix patriciarum*, and *Piromyces communis* can digest cereal starches [29]. However, the presence of protozoa and fungi for starch degradation in the rumen is not essential.

Pectin can be fully digested by bacteria and protozoa, and its degradation rate in the rumen is higher than that of other carbohydrates. Pectin-degrading bacteria include *B. fibrisolvens*, *P. ruminicola*, *L. multipara*, *S. bovis*, *Succinivibrio dextrinosolvens*, and *Treponema saccharophilum* [30]. Additionally, the common cellulolytic bacteria *R. albus* and *F. succinogenes* can degrade pectin [31]. Among them, the primary pectin-degrading bacteria are *B. fibrisolvens* and *Prevotella* species [32]. Notably, similar to ciliate protozoa, *S. bovis* can degrade pectin but cannot utilize the degradation products.

#### Microbial species related to protein degradation in the rumen

This protein degradation process involves the participation of many bacteria, and proteolytic and peptidolytic bacteria account for approximately 65% of rumen bacteria [33]. The most important proteolytic bacteria are *B. fibrisolvens* and *B. proteoclasticus* [34], which exhibit high proteolytic capacity, and the abundance of *B. fibrisolvens* increases when animals are fed high-protein diets [35]. Additionally, *R. amylophilus*, *E. budayi*, *Streptococcus bovis*, *Selenomonas ruminantium* [36], and other bacteria also exhibit proteolytic capabilities. While these bacteria may be present at lower abundances in the rumen, they play a crucial role in protein metabolism due to their high proteolytic abilities. *Prevotella* species, such as *P. albensis*, *P. brevis*, and *P. bryantii*, also possess proteolytic capabilities [37]. Although their proteolytic capacity may be lower than that of other bacteria, these species are important contributors to protein degradation due to their higher abundance in the rumen. Protozoa contribute to approximately 20% of the proteolytic activity in the rumen [38]. Protozoa such as *Entodinium caudatum*, *Entodinium simplex*, *Dasytricha ruminantium*, and *Polyplastron multivesiculatum*, exhibit proteolytic abilities [39]. Studies have isolated the fungus *Neocallimastix frontalis* from sheep rumen, which exhibits high proteolytic activity and plays an important role in rumen protein degradation [40]. However, other studies comparing the activities of rumen proteolytic fungi suggest that the capacity of these fungi for protein hydrolysis is limited.

Peptide degradation is an intermediate step in protein breakdown, and *Prevotella* species exhibit broad peptidolytic activity. Among these, *P. ruminicola* stands out as a pivotal species for peptide degradation in the rumen, exhibiting a dipeptidyl peptidase range and specific activity that surpasses those of other prevalent peptidolytic bacteria [37]. Additionally, *P. albensis* and *P. bryantii* possess peptidase activity. Apart from *Prevotella* species, *S. bovis*, *R. amylophilus*, *Veillonella parvula*, *Ruminococcus* species, *Megasphaera elsdenii*, *L. multipara*, *F. succinogenes*, and *E. ruminantium* [41] exhibit weaker peptidase activity, contributing less to peptide degradation in the rumen. In the absence of bacteria, ciliate plays a significant role in the accumulation and breakdown of dipeptides [42], for example, *Entodinium* species, *Dasytricha ruminantium*, and *Isotricha* species can degrade oligopeptides in the rumen.

#### Microbial species related to lipid degradation in the rumen

Lipids in the rumen are hydrolyzed into galactose, glycerol, and long-chain or medium-chain fatty acids. The first identified lipid-degrading bacterium in the rumen was *Anaerovibrio lipolyticus* [43], followed by the isolation of various other lipid-degrading bacteria, including *B. fibrisolvens*, *Clostridium* species, and *Propionibacterium* species [44]. The prominent lipid-degrading bacteria include *A. lipolytica* and *B. fibrisolvens*. Different lipid-degrading bacteria exhibit varying abilities and preferences for specific types of lipids. For instance, *B. fibrisolvens* can only degrade polar lipids, while *Propionibacterium* species can only degrade neutral lipids [44]. There is limited research on fungi and protozoa involved in lipid degradation. Some studies have found that the protozoan species *Entodinium caudatum* has phospholipase activity, but its relevance to dietary lipids remains uncertain [45]. Reports suggest that the protozoa *Epidinium* species account for 30% to 40% of the lipid degradation activity in the rumen, but it is generally believed that bacteria are the primary species responsible for lipid degradation [46]. In the rumen, the breakdown of unsaturated fatty acids involves their hydrogenation into saturated fatty acids. This process may serve as a detoxification mechanism because unsaturated fatty acids are more toxic to microorganisms than saturated fatty acids [47]. Bacteria and protozoa participate in the process of biohydrogenation, and these bacteria include *B. hungatei*, *B. proteoclasticus*, *Propionibacterium acnes*, *E. ruminantium*, *C. proteoclasticum*, *Pseudobutyrivibrio* species [44,48]. These bacteria can be divided into the following groups: one can convert linoleic acid to trans-11-octadecenoic acid, and the other produces stearic acid as the end product.

### Rumen microbial species involved in the fermentation processes

Rumen microorganisms primarily ferment soluble sugars, amino acids, and glycerol to generate products such as VFAs. The majority of the rumen microbiome, including the genera *Streptococcus*, *Bifidobacterium*, *Lactobacillus*, *Treponema*, *Selenomonas*, *Veillonella*, *Coprococcus* and *Megasphaera* [6, 49], can ferment these soluble sugars. Among them, *S. bovis* and *Lactobacillus* species are important, as they can rapidly proliferate in the presence of excess carbohydrates [48]. Succinate, lactate, and fumarate are intermediate products in the fermentation process and eventually convert into VFAs. One of the pathways is called the succinate pathway, which involves the reduction of pyruvate to produce succinate and its subsequent conversion to propionate. Bacteria from the phyla Firmicutes and Bacteroidetes are involved in the succinate pathway [6]. Specifically, bacteria such as *Actinobacillus succinogenes* and *Mannheimia succiniciproducens* are responsible for succinate production in the rumen [50], while *Succiniclasticum ruminis* is among the succinate-utilizing bacteria [51]. Another pathway, the acrylate pathway, converts lactate into propionate, among others. This pathway is vital in the rumen, as it prevents the accumulation of lactate, which can lead to acidosis due to a decrease in ruminal pH. Common lactate-utilizing bacteria include *S. ruminantium* and *M. elsdenii*, which ferment lactate to acetate and propionate [52]. Bacteria from the genera *Lactobacillus*, *Streptococcus*, *Enterococcus*, and *Pediococcus* are primarily responsible for lactate production.

Most of the amino acids are rapidly fermented in the ru men; the first step produces ammonia and keto acids, which are then converted into VFAs. Almost all proteolytic bacteria are involved in the deamination process. Deaminating bacteria can be divided into two main categories. The first category includes bacteria with lower deamination capability but high abundance in the rumen [53], such as *B. fibrisolvens*, *P. ruminicola*, *M. elsdenii*, and *Allisonella histaminiformans*. The second category consists of bacteria with strong deamination capability but low abundance, also known as high-ammonia-producing bacteria. These bacteria include *C. aminophilum*, *C. sticklandii*, and *Peptostreptococcus anaerobius*. Notably, high-ammonia-producing bacteria cannot participate in protein degradation and can only ferment amino acids as their only nitrogen source. Glycerol broken down from lipids is rapidly fermented in the rumen into VFAs, CO_2_, and H_2_. *A. lipolytica*, *B. fibrisolvens*, and *S. ruminantium* are involved in this fermentation process [48].

### Rumen microbes involved in methane production

The hydrolysis and fermentation processes of macromolecules generate a large amount of hydrogen, which can be converted into methane in the rumen. Methanogens are considered a key driving force in the entire food chain [54]. Methanogens that have been cultured from rumen contents include *Methanobacterium formicium*, *Methanobacterium bryantii*, *Methanobrevibacter olleyae*, *Methanobrevibacter millerae*, *Methanobrevibacter ruminantium*, *Methanomicrobium mobile*, *Methanoculleues olentangyi*, and *Methanosarcina barkeri* [48]. Among them, the most prevalent methanogen genus was *Methanobrevibacter*, constituting 66% to 68% of the archaeal population [55]. The most distinctive methanogens are *Methanobrevibacter ruminantium* [48], and *Methanobrevibacter gottschalkii*, and *Methanobrevibacter ruminantium* are generally dominant.

Methanogenic archaea can be classified into the following types based on different substrates: H_2_/CO_2_ (hydrogenotrophic), methane derivatives (methylotrophic), and acetate (acetoclastic). The hydrogenotrophic pathway is the main route for methane production, with approximately 78% of methanogens participating in this process [49]. The most important hydrogenotrophic methanogen genera are *Methanobrevibacter*, *Methanosphaera*, *Methanimicrococcus*, and *Methanobacterium* [56]. The methylotrophic pathway involves the simultaneous utilization of methyl compounds, for growth. Approximately 22% of methanogens are participate in the methylotrophic process. The archaea mainly involved in methylotrophy belong to the order *Methanosarcinales*, *Methanococcoides*, *Methanosarcina*, and *Methanolobus* and are the major methanogens involved in the methylotrophic process [57]. Compared to hydrogenotrophic methanogenesis, acetoclastic methanogenesis is less common in the rumen. The low abundance of these archaea may occur because their growth rate is slower than the acetate production rate [48]. The production of methane is primarily conducted by methanogenic archaea under anaerobic conditions. However, recent research has revealed that eukaryotes (including plants, animals, and fungi) can also actively participate in methane production in the presence of oxygen [58]. The methane emission strategies in the rumen target the above methanogens and the key enzymes in the methane production pathways. Balancing methane production and hydrogen retention is essential for achieving emission and normal fermentation by exploring the holistic function of the rumen microbial system.

Many microbial species involved in the above common functions within the rumen have been successfully cultured, which expands our knowledge on rumen biology and nutrition. These cultured species offer potential regulated targets to enhance the basic but important functions in the degradation of feed and contribute to the components of probiotics used for improving rumen digestion or feed digestibility. Based on the Hungate 1000 project, approximately 500 cultured rumen microbial species are currently available contributing to only 3.7% of the estimated total microbial numbers in the rumen [49,59]. Culture-independent approaches, such as meta-omics technologies are urgently needed to unravel the extensive functions in the rumen.

## APPLICATION OF META-OMICS ON RUMEN MICROBIAL FUNCTIONAL STUDIES

### Using metagenomics to reveal potential functions

The concept of the metagenome was proposed by Handelsman et al [60] for the first time, and this concept refers to the sum of all microbial DNA in a specific environment. Metagenomics can sequence all microbial genetic material DNA in the sample, eliminating the problem that most microorganisms in the environment cannot be cultured [60]. Genes with potential functions in microorganisms can be obtained by open reading frame (ORF) prediction and functional annotation. Early research on rumen metagenomics mainly focused on exploring the genes encoding carbohydrate-active enzymes, especially the genes encoding lignocellulases that degrade plant cell walls. In 2009, Brulc et al [61] investigated carbohydrate-active enzymes in the rumen of three beef cattle fed the same diet. A total of 35 glycoside hydrolase genes were found, but only three carbohydrate-binding enzyme-encoding genes and three anchor modules were found. Although only three animals were used in this study, this study was the first to use metagenomic sequencing techniques to define a fiber adhesion microbial community. Later, metagenome sequencing was used to explore the functional genes of rumen microbial degradation by Hess et al [4]. The study increased the amount of metagenome sequencing data to 2.5 million ORFs, of which approximately 1% were identified as carbohydrate-active genes. Most of the genes were inconsistent with the NCBI nonredundant database, indicating that the rumen microbiome contains a wide range of fiber-degrading enzyme types. Ninety genes were selected for expression, 57% of which encoded enzymes with cellulolytic activity. In other ruminants, Pope et al [62] and Dai et al [63] reported the rumen metagenome of yaks and reindeer and also explored the genes encoding carbohydrate-active enzymes.

Metagenomic sequencing technology can also investigate the impact of methane inhibitors on rumen microbiota and their functionality, identifying key bacteria that regulate methane production. Ross et al [64] found that two distinct methane-reducing feed additives altered the microbial composition of samples in a similar manner, and from this result, they identified *Faecalibacterium* species as a potential biomarker for low methane-emitting cattle. Denman et al [65] discovered that inhibition of methanogens by bromochloromethane (BCM) directly and indirectly impacted the rumen microbiome. Among them, the relative abundance of hydrogen-utilizing bacteria such as *Prevotella* and *Selenomonas* species increased, resulting in the production of more propionate and suppression of methane generation. Later, a large number of studies on the methane emission of ruminants focused more on the natural selection of high- and low-methane emission animals using metagenomic technology. In beef cattle, methanogens and their genes are more abundant in the rumen of animals with high methane emissions [66,67]. Auffret et al [68] fed different diets to a group of beef cattle from different breeds to determine the rumen metagenome. It was found that the abundance of the methane generation pathway was strongly related to methane emissions, while the abundance of methanogens was weakly related to methane emissions. Shi et al [69] and Kamke et al [70] reported the relationship between rumen microbes and methane emission in goats using metagenomics. In addition to methane emissions, some metagenomics studies have also focused on beef cattle and cows [71,72], and reported the relationship between rumen microbial flora and function and feed utilization efficiency.

### Using metagenome-assembled genomes to reveal potential functions of single microbes

With the innovation and development of high-throughput sequencing technology and bioinformatics analysis tools, an increasing number of studies are using metagenome-assembled genomes (MAGs) for genomic analysis. In comparison to conventional metagenomic analysis, MAGs involve an additional step called metagenomic binning. This process involves categorizing the mixed sequences obtained from metagenomic sequencing or contigs assembled from the sequences into separate groups based on their respective species. MAGs with high completeness and low contamination levels were used to perform further taxonomic annotation and gene prediction [73]. MAGs are advantageous because they can overcome the limitations of reference genomes, including their availability and completeness. Metagenome assembly and binning can be performed *de novo*, enabling the discovery of new or uncultivable microorganisms [74]. Additionally, MAGs, through assembly, can identify short genes that might be missed by gene-finding tools; these short genes can be missed due to their small open reading frames (sORFs), which are a common feature of all genomes and hold significant untapped coding potential [75]. Due to these advantages, a limitation of conventional metagenomic analysis is addressed, making these tools broadly applicable to studies on the rumen microbiota.

As early as 2011, Hess et al [4] based on 268 G rumen metagenome data, successfully binned the genomes of 15 noncultivable microorganisms and verified them by single-cell whole-genome sequencing. Subsequently, researchers successively constructed the rumen MAGs of different ruminants. For example, 43 Scottish cattle, 913 bacterial and archaeal MAGs were assembled using more than 800 G of metagenomics data [5]; most of these strains had never been sequenced. In total, 69,000 proteins involved in carbohydrate metabolism were predicted, of which more than 90% had not been matched in the public database. Xie et al [76] first constructed a gene catalog of the entire gastrointestinal tract microbiota in ruminants, obtaining over 10,000 MAGs. This effort led to the identification of nearly 9,000 potentially novel bacteria and archaea, significantly expanding the known diversity and functions of the gastrointestinal microbiota in ruminant animals. MAGs are widely used to explore specific metabolic mechanisms of the rumen microbiota. Jiang et al [77] analyzed 17,000 gastrointestinal microbial genomes (10,373 MAGs from a previous study and 7,052 genomes from the collection of public ruminant microbial genomes). The researchers identified 2,366 high-quality genomes involved in the biosynthesis of vitamins B and K2. This study demonstrated regional heterogeneity and dietary effects on the potential for vitamin biosynthesis within the gastrointestinal microbiota of ruminant animals. In another study by Lin et al [78], they obtained 372 MAGs involved in bile acid (BA) transformation pathways were obtained from 108 samples of the entire gastrointestinal contents of 18 cows, revealing the rumen microbial BA metabolism mechanisms.

However, MAGs still have certain limitations, such as gaps, local assembly errors, chimeras, and contamination from other genomic fragments, which can restrict the value of these genomes [79]. These errors are often caused by immature sequencing technologies and bioinformatics algorithms. As sequencing depth increases and assembly techniques continue to improve, the quality of MAGs should be significantly improved. For instance, the high-quality sequencing technology of HiFi reads introduced by PacBio can enhance the completeness and accuracy of assembled genomes [80], and the development of assembly validation tools has played a crucial role in improving metagenome assembly [81]. Furthermore, there are still numerous uncharacterized microorganisms, and a comprehensive and well-curated reference gene database is needed for comparison and identification.

### Using metatranscriptomics to reveal potential active functions

Although the rapid development of metagenomics technology has greatly enriched our knowledge on the rumen microbial diversity and function of ruminants, metagenomics still has some limitations. For example, metagenomics fails to reflect the real activity and functional characteristics of rumen microorganisms [82]. To investigate the composition of the active microbiota and the expression of active genes within the rumen microbiota at a specific time and space in situ, metatranscriptomics techniques are essential. Metatranscriptomics is a technique used to study the whole genome transcription and transcriptional regulation of microbial populations in a certain time and space, reflecting the true state of the rumen microbial community at the transcriptional level [83]. Compared with metagenomics, metatranscriptomics has been relatively slow to develop and has been applied to the study of rumen microorganisms with relatively few applications. A significant limitation is that RNA has a half-life period and tends to degrade during storage; thus, compared to total DNA, total RNA is much more challenging to extract total RNA than total DNA from rumen microorganisms.

Since most of the previous RNA sequencing analyses involved eukaryotic mRNA, the first large-scale rumen metatranscriptomics analysis also focused on rumen eukaryotes. Qi et al [84] used metatranscriptome technology to investigate the functional diversity of eukaryotic microorganisms within the rumen, revealing a significantly higher percentage of cellulase enzymes compared to metagenomics. The metatranscriptome can be a suitable approach to identify potential gene targets. Since then, an increasing number of studies on fiber degradation have employed metatranscriptomics technology. Dai et al [85] removed the rRNA from the total RNA and sequenced a total of 1 million nonrRNA sequences, of which approximately 1% were identified as carbohydrate-active enzymes or binding modules. In another cow study, similar levels of carbohydrate genes were obtained by Shinkai et al [86] using mRNA-enriched metatranscriptome sequencing data. The above studies confirmed that the main active bacteria responsible for fiber degradation were *Fibrobacteraceae* and *Clostridiaceae*. A cow study in 2017 used 18 new ribosome capture probes covering a large number of rumen archaea, bacteria, fungi, and prokaryotes, which confirmed that the bacteria could degrade fiber; in addition, fungi and protozoa greatly contributed to fiber degradation [87].

In terms of methane emission, the researchers used the rRNA and mcrA libraries to study rumen methanogens in the early stage and found a group of archaea similar to Thermoplasmatales. Because these microbes are ubiquitous in the rumen and encode mcrA genes, they may be methanogenic archaea [88]. However, before metatranscriptomics, a clear link between methanogenic capacity and these microorganisms could not be established. According to transcriptomics results, Poulsen identified a new cluster of methylotrophic methanogens [3]. In sheep, metatranscriptomics analysis revealed clear differences in rumen methanogens and related metabolic pathways between animals with low and high methane emissions, revealing a correlation between hydrogen trophic methanogens and methane production. However, based on metagenomics, no similar difference was found, which may result from the hydrogen supply of other rumen microbial fermentation pathways [69]. Following this study, metatranscriptomics was used to explore bacteria [70]. Based on this study, bacteria that were fermented into lactate and subsequently refermented into butyrate salts would decrease hydrogen production, thereby reducing methane generation.

### Using metaproteomics to reveal functional proteins

Protein stands out as a direct and pivotal embodiment of microbial gene function. Therefore, research on protein composition and function based on the metagenome will help researchers study the abundance and distribution of functional molecules in microbial populations. Rumen metaproteomics describes the gene expression protein of the rumen microbial community in a specific time and space. Hart et al [89] compared the metaproteomics results with the protein functions predicted by metatranscriptomics and found that only 71% of the metaproteomics information matched the metatranscriptomics data, which indicated that metaproteomics could more accurately reflect the expression of environmentally active microorganisms than metatranscriptomics. Moreover, compared with the metagenome, the metaproteome is a more reliable indicator of animal phenotypes and achieves more accurate classifications [90]. As a result, metaproteomics plays a pivotal role in the study of microbial community function, and its advantages are attracting increasingly the attention of scientists. However, the method used to analyze metaproteomics bioinformatics still needs to be improved urgently. Due to the particularity of the research object, the analysis of metaproteomics requires a different set of bioinformatics and statistical models from those of traditional proteomics [91]. Therefore, biological meaning behind metaproteomics big data can only be determined once these problems are solved. With the increase and development of the next- and third-generation sequencing technology, and the significant reduction in sequencing costs, large-scale microbial sequence information is constantly being revealed, and the microbial genome database is also gradually improving; as a result, the protein identification method, speed, and accuracy of metaproteomics are promoted and will be significantly improved.

At present, there are relatively few applications of meta proteomics to rumen microorganisms. In an earlier study of rumen microbes in sheep, researchers attempted to identify cellulose-binding proteins through enrichment steps [92]. In this study, MS/MS-1D polyacrylamide gel electrophoresis (PAGE) was used to identify a small number of proteins and to link these proteins with microbial species. However, the limited database at that time restricted data mining. The proteins identified in this study include endoglucanase from *F. succinogenes* and exoglucanase from the fungus *Piromyces equi*. The combination of 2D PAGE separation technology and liquid chromatography-tandem mass spectrometry (LC–MS/MS) improves the resolution and facilitates the discovery of more peptides [93]. In this study, it was found that the enzyme of methanogens is among the most easily identified proteins, suggesting that metaproteomics may play an important role in exploring the rumen mechanism related to methane emission in ruminants. Recently, using metaproteomic analysis [94], the effects of digestion and methane metabolism in the rumen of ciliates have recently been elucidated. The shotgun metaproteome method can generate a much larger amount of data and offers a potential alternative to gel-based methods. Deusch and Seifert [95] were the first to employ the shotgun metaproteome method to identify prokaryotic and eukaryotic proteins in plant-attached microorganisms and rumen contents, showcasing a significant improvement in protein identification ratios. Subsequently, Deusch et al [96] conducted more complex explorations, identifying over 8,000 bacterial proteins and 350 archaeal proteins. These researchers also detected a substantial number of proteins involved in carbon metabolism.

### Using metabolomics to reveal metabolic potentials

Metagenomics, metatranscriptomics, and metaproteomics are used to study the life activities at the levels of genes, transcription, and proteins, respectively. Many of the biological activities in cells occur at the level of metabolites. For example, cell signal release, energy transmission, and intercellular communication are regulated by metabolites. Metabolomics is used to perform qualitative and quantitative analysis of all metabolites in an organism or a cell at a specific time and space [97]. The research objects of metabolomics are mostly small molecules with a relative molecular weight less than 1,000 [98]. Compared with metagenomics, metatranscriptomics, and metaproteomics, metabolomics can more easily detect and amplify small changes in gene and protein expression, making detection easier; in addition, stronger versatility and nonspecificity of metabolites are observed in various tissues. The results are more direct, and the metabolites can reflect the physiological and case status of the biological system. Targeted metabolomics determination commonly used in traditional ruminant nutrition research includes quantitative determination of a limited number of VFAs and quantitative determination of methane and hydrogen content [99,100]. Currently, nontargeted metabolic measurements are also widely used to study rumen microbial metabolomics [101], among which GC–MS and LC–MS are widely applied.

Metabolomics studies on ruminants often examine how the ratio of concentrate to roughage affects rumen metabolites, making it a significant area in ruminant metabolomics. Ametaj et al [101] and Saleem et al [100,102] conducted investigations on rumen metabolism changes with increasing proportions of concentrate in the diet. A variety of detection methods were used to identify rumen metabolites, and 246 of these metabolites were identified, mainly including phospholipids, inorganic ions, gases, amino acids, short-chain fatty acids, and carbohydrates. Combined with 87 metabolites reported in the literature, a database of rumen metabolites was created. In addition, other studies have reported changes in rumen metabolic patterns caused by changes in the ratio of concentrate to crude in the diet. For example, it was found that an increase in dietary cereals could lead to an increase in rumen methylamine content and a decrease in 3-phenylpropionate. Additionally, differences in the efficiency of feed utilization in ruminants can be reflected in rumen metabolites. The notable observation indicates the correlation between the metabolism of the rumen biological hydrogenation pathway (encompassing linoleic acid and alpha-linoleic acid) and average daily gain [99]. In cows, the increase in rumen short-chain fatty acid content and putrescine content and the decrease in methane content are related to high feed utilization efficiency [71].

Unlike other omics methods, metabolomics cannot directly link metabolites with microbial communities. Hence, the method must be combined with microbial relative abundance data obtained by other omics technologies for an integrated analysis. For example, in reports related to methane emissions, researchers found that changes in urine and plasma metabolites (trimethylamine N-oxide) were related to species of rumen protozoa and *Methanomassilii cocus*, indicating that trimethylamine N-oxide could be an important means of reflecting methane emissions [103,104]. In addition, the combined analysis of multiomics including rumen metabolomics effectively reflects the functional level information of nonculturable rumen bacteria. For example, by using metagenomics data to determine several genomes of the uncultured *Bacteroides BS11* family, researchers subsequently validated their functions through metaproteomics and metabolomics data, which were identified it as crucial executors of hemicellulose degradation [105]. The combination of metabolomics with other omics technologies shows great potential to reveal mechanisms underlying ruminant diseases.

## NEW OPPORTUNITIES

Research on the rumen microbiota has predominantly focused on rumen bacteria and archaea, but an increasing number of studies are focusing on other microorganisms present in the rumen. For example, rumen ciliates play a crucial role in the rumen environment, but their specific metabolic functions have remained unclear due to their nonculturable nature. Li et al [106] published the first catalog of rumen ciliate genomes, uncovering new ciliates and revealing their remarkable ability to degrade plant cell walls. Through the analysis of metaproteomic data from rumen samples, Andersen et al [94] provided a detailed description of specific metabolic niches occupied by ciliates in their microbiome environment, highlighting their significant impact on digestion and methane metabolism. In marine environments, viruses are driving factors in nutrient and energy cycling. However, research on the community of rumen viruses currently lags behind other topics. Using viral metagenome sequencing, Anderson et al [107] discovered that rumen viruses can breakdown complex carbohydrates and significantly influence microbial metabolism. Using proteomics, Solden et al [108] identified phages as active regulators of rumen ecosystem functionality. In terms of gene functionality, 50% to 70% of rumen viral reads possess viral replication capabilities, while other reads exhibit functional diversity. Auxiliary metabolic genes (AMGs) represent a subset of viral genes that redirect host metabolism toward reactions favorable for phage replication. Therefore, AMGs may reflect the potential impact of rumen viruses on microbial community metabolism. Nonetheless, there are still numerous unknown viral genes that must be further investigated. Omics technologies assist in gaining knowledge on viruses and their interactions. However, omics technologies still face some unresolved issues, such as limitations in the use of DNA sequencing methods to study RNA-based viruses and the predominance of phages in public reference databases, which potentially hinder the identification of archaeal viruses and others. Apart from ciliates and viruses, fungi and protists have emerged as new areas of research interest.

A multitude of emerging technologies are currently revolu tionizing our knowledge on microbiome functions. Organoids, which are self-organizing 3D tissues, can to mimic the intricate functions, structures, and biological complexity of organs. Specifically, intestinal organoids exhibit the complexity needed to replicate physiological and pathological conditions related to diet, microbiota, and host interactions, shedding light on the mechanisms governing microbial-related functions [109]. The concept of the “Ramanome” involves compiling multiple single-cell Raman spectra from cell populations at specific conditions and time points. As demonstrated by Jing et al [110], the Ramanome approach has been successfully utilized to identify specific functional bacteria isolated from seawater. Raman spectroscopy, through which individual microbial cells can be analyzed in a nondestructive manner, allows for further cell cultivation or DNA analysis, ultimately improving our knowledge on their functional activities. Through applying these emerging technologies, we can gain comprehensive insights into the intricate roles of microorganisms, making it possible to effectively target and regulate the physiological functions of animal organisms by manipulating the rumen microbiota.

Through metagenomics and other techniques, we have clarified the functionality of rumen microbiota to further manipulate them. Santra et al [111] suggested that digestive functions can be enhanced and nutrition and productivity can be optimized by manipulating these microorganisms. Currently, various methods are used to manipulate rumen microbiota, which occurs primarily through dietary control, such as the use of chemical additives, direct microbial supplementation, and probiotics [112]. However, the effects of dietary control on the adult rumen microbiome and fermentation are typically effects. Additionally, some methods involve the transfection of rumen microbiota, in which microbial communities are physically introduced into the cow’s rumen; however, these transplanted microorganisms do not seem to persist [54]. Therefore, extensive research is still needed to clarify the functionality of the rumen microbiota and determine how to manipulate it effectively. Current approaches primarily focus on manipulating heritable microorganisms through breeding programs, as these microorganisms are vital constituents of the symbiotic network within the rumen microbiota, potentially exerting pleiotropic effects on microbial composition [113]. Another approach is early-life interventions, which may generate long-term effects on rumen function [114]. However, the optimal time for producing sustained effects by manipulating the rumen microbiota remains to be determined.

## CONCLUSION

Through the collective action of the rumen microbiota, high-fiber feed that monogastric animals cannot digest can be degraded and fermented. This process serves as an energy source and a microbial protein source for ruminant animals. However, it also results in the production of significant amounts of methane. Numerous omics technologies have been employed to characterize the rumen microbiota, continuously seeking fresh insights into the functionality of these complex microbial communities. Partitioning microbial communities into functional groups based on metabolic functions provides a more precise assessment of the community’s status and functions, addressing limitations associated with exclusive reliance on species taxonomy, including vagueness and redundancy. Integrating taxonomy with functional groups enhances our comprehensive understanding of the rumen ecosystem’s functions, thereby facilitating the design of interventions within the rumen microbiota. As a result, the performance of the rumen ecosystem can be further improved.

Hence, through exploring microbial communities more comprehensively with advanced and innovative omics techniques, the rumen microbiota can be more easily manipulated through diet or other means, improving the production efficiency of ruminant animals, reducing methane emissions, and providing more possibilities for the future of sustainable livestock farming.

## Figures and Tables

**Table 1:** Classification of major rumen functional bacteria

Functional classification	Species	Reference
Cellulolytic bacteria	*Fibrobacter succinogenes*, *Ruminococcus flavefaciens*, *Ruminococcus albus*, *Clostridium lochheadii*, *Clostridium longisporum*, *Butyrivibrio fibrisolvens*, *Eubacterium cellulosolvens*	[12], [14], [15]
Hemicellulolytic bacteria	Cellulolytic bacteria, *Lachnospira multiparus*, *Prevotella* sp., *Pseudobutyrivibrio xylanivorans*, *Streptococcus* sp.	[19], [20]
Amylolytic bacteria	*Fibrobacter succinogenes*, *Streptococcus bovis*, *Prevotella ruminicola*, *Clostridium* sp., *Ruminobacter amylophilus*, *Succinimonas amylolytica*, *Ruminococcus bromii*, *Selenomonas ruminantium*, *Butyrivibrio fibrisolvens*	[22], [23], [24]
Pectin-degrading bacteria	*Fibrobacter succinogenes*, *Ruminococcus albus*, *Butyrivibrio fibrisolvens*, *Streptococcus bovis*, *Treponema* sp., *Cellulosilyticum ruminicola*, *Lachnospira multipara*, *Prevotella* sp.	[30], [31], [32]
Proteolytic bacteria	*Butyrivibrio fibrisolvens*, *Butyrivibrio proteoclasticus*, *Streptococcus bovis*, *Prevotella* sp., *Ruminobacter amylophilus*, *Eubacterium budayi*, *Selenomonas ruminantium*	[34], [36], [37]
Peptidolytic bacteria	*Fibrobacter succinogenes*, *Prevotella* sp., *Ruminococcus* sp., *Eubacterium ruminantium*, *Streptococcus bovis*, *Ruminobacter amylophilus*, *Veillonella parvula*, *Megasphaera elsdenii*, *Lachnospira multipara*	[37], [41]
Lipid-degrading bacteria	*Anaerovibrio lipolytica*, *Butyrivibrio fibrisolvens*, *Clostridium* sp., *Propionibacterium* sp.	[43], [44]
Biohydrogenating bacteria	*Butyrivibrio hungatei*, *Butyrivibrio proteoclasticus*, *Propionibacterium acnes*, *Eubacterium ruminantium*, *Clostridium proteoclasticum*, *Pseudobutyrivibrio* sp.	[44], [48]
Lactic producing bacteria	*Bifidobacterium lactis*, *Lactobacillus acidophilus*, *Streptococcus bovis*	[52]
Lactic utilising bacteria	*Selenomonas ruminantium*, *Megasphaera elsdenii*	[52]
Succinate producing bacteria	*Actinobacillus succinogenes*, *Mannheimia succiniciproducens*	[50]
Succinat utilising bacteria	*Succiniclasticum ruminis*	[51]
Deaminating bacteria	*Butyrivibrio fibrisolvens*, *Prevotella ruminicola, Megasphaera elsdenii*, *Allisonella histaminiformans*, *Clostridium aminophilum*, *Clostridium sticklandii*, *Peptostreptococcus anaerobius*	[53]
Glycerol fermenting bacteria	*Anaerovibrio lipolytica, Butyrivibrio fibrisolvens, Selenomonas ruminantium*	[48]
